# Preparation of Mesoporous Si Nanoparticles by Magnesiothermic Reduction for the Enhanced Reactivity

**DOI:** 10.3390/molecules28073274

**Published:** 2023-04-06

**Authors:** Xinwen Ma, Weiduo Fei, Xiandie Zhang, Jie Ji, Xiang Zhou

**Affiliations:** School of Chemistry and Chemical Engineering, Nanjing University of Science and Technology, Nanjing 210094, China

**Keywords:** energetic materials, mesoporous silicon nanoparticles, magnesiothermic reduction, thermal analysis

## Abstract

In this study, mesoporous silicon nanoparticles (M-Si) were successfully prepared by a magnesiothermic reduction of mesoporous silica nanoparticles, which were synthesized by a templated sol-gel method and used as the precursors. M-Si exhibited a uniform size distribution with an average diameter of about 160 nm. The measured BET surface area was 93.0 m^2^ g^−1^, and the average pore size calculated by the BJH method was 16 nm. The large internal surface area provides rich reaction sites, resulting in unique interfacial properties and reduced mass diffusion limitations. The mechanism of the magnesiothermic reduction process was discussed. The reactivity of prepared M-Si was compared with that of commercially available non-porous Si nanopowder (with the average diameter of about 30 nm) by performing simultaneous thermogravimetry and differential scanning calorimetry in the air. The results showed that the reaction onset temperature indicated by weight gain was advanced from 772 °C to 468 °C, indicating the promising potential of M-Si as fuel for metastable intermolecular composites.

## 1. Introduction

The introduction of nanotechnology into the design of energetic materials has resulted in a new type of highly reactive material: metastable intermolecular composites (MICs) [1,2,3]. Accompanied by significant nano-scale effects, MICs have a more uniform spatial distribution as well as enhanced interfacial contacts, along with tunable energy release and combustion properties [4,5,6,7]. Therefore, MICs are full of promising applications in the fields of detonation [8], micro-propulsion [9], material processing and synthesis [10], etc.

Currently, the fuel sources mainly include metals (aluminum (Al) [11], magnesium (Mg) [12], titanium (Ti) [13], etc.) and metalloids (boron (B) [14], silicon (Si) [15], etc.). In addition, most of the studies on MICs have focused on Al as the fuel to explore its reaction and exothermic properties when combined with different oxidants. However, Al powders are very sensitive to electrostatic discharge, friction, and mechanical shock. Moreover, the oxide layer of ~2–6 nm will directly lead to the loss of active content, especially when Al powders reach the nanometer scale. Additionally, the Al core cannot diffuse easily through the dense oxide shell unless the internal Al core is melted or the oxide shell has undergone a phase change [16,17]. Among other fuel options, Si is an attractive one because of the thin oxide layer and the insensitivity to accidental stimuli. Moreover, the thermodynamic enthalpy of the oxide formed by elemental Si is comparable to that of aluminum on a per weight basis (15.2 kJ g^−1^ and 16.6 kJ g^−1^, respectively) [18]. However, Si is less reactive than Al, which can limit its wide applications. It was reported that in order to obtain the available energy, the reaction rate must be fast enough during the expansion of the explosion products in the early period [19].

It is undeniable that the lack of silicon reactivity limits its further applications, and how to enhance the reactivity of silicon has become an urgent problem to be solved for carrying out silicon-based MIC applications. A lot of work has been done by previous researchers in exploring strategies to improve the reactivity of silicon. Huang et al. prepared Si/Fe_2_O_3_ nanothermites with a core-shell structure using a simple electroless plating method, and the reaction onset temperature of ~550 °C is almost comparable to that of the commonly used Al-based nanothermites, such as Al/Fe_2_O_3_, Al/CuO, and Al/Co_3_O_4_, etc. [17]. Cole et al. investigated the effect of low levels of lattice doping in silicon on the combustion and reaction kinetics of Si/PTFE and found that the apparent activation energy and combustion rate of Si/PTFE showed a positive correlation with the dopant concentration, which in turn could further tune the reaction properties of silicon-based energetic powders [20]. Zhao et al. doped titanium (Ti) nanoparticles with a lower onset oxidation temperature than that of Si into the Si/KClO_4_ system, and the lower ignition temperature of Ti/KClO_4_ led to the initiation reaction of the ternary composites, thus promoting energy release and the reactivity of the ternary system [13].

Apparently, the reduction of particle size to the nanometer scale is a straightforward way to boost reactivity [21,22,23]. However, continuing size reduction is of limited effect due to the inevitable agglomeration, which poses a huge challenge for practical applications. As an alternative, creating porous nanoparticles allows for simultaneous structural design and size effect, with the large internal surface area providing richer reactive sites and bringing unique interfacial properties and reduced mass diffusion limitations [24]. In addition, the detectable Si–H_x_ bonds seem to play a key role in the low-temperature explosion behavior of porous silicon [25]. Since the observation of highly exothermic reactions between porous silicon and nitric acid or liquid oxygen [26], researchers have explored the filling of various oxidants as well as corresponding exothermic behavior, particularly with elemental sulfur, which can be fused directly into the pores in the absence of a solvent [27,28]. In the work of Parimi et al., a phenomenological model describing the interaction between silicon and oxidant in a single nanoscale pore was developed, which showed that the reactive wave propagation is more controlled by the specific surface area than the global equivalent ratio due to the diffusion length scale. More reactive crystal surfaces and a higher surface area bring higher propagation slightly faster, thus elucidating the effect of the fuel–oxidant interface [29].

In the past years, the preparation of porous silicon has mainly relied on the etching method. The pore size of porous silicon nanoparticles prepared by electrochemical etching is small and uniformly distributed [30,31]. However, the special physical morphology of silicon wafers limits the development of a wide range of applications in the field of energetic materials. Although its powders could be obtained from anodically oxidized porous silicon wafers by certain separation means, this method is only applicable in the field of low-volume, high-value products such as drug delivery [32]. The stain-etching technique is low cost and simple to operate, but it generates toxic volatile gases and is poorly reproducible due to the large amount of HF and concentrated HNO_3_ used in the experimental process [33]. The magnesiothermic reduction method not only allows the product to maintain the same morphology as the precursor but also significantly reduces the reduction temperature compared to the industrialized carbonothermic reduction [34].

In this study, to investigate the effect of porous structures on the reaction properties of silicon, we first prepared mesoporous silica nanoparticles (M-SiO_2_) with high porosity using a one-pot method, and then we obtained mesoporous silicon nanoparticles (M-Si) using a molten salt-assisted magnesiothermic reduction process. DSC–TG tests showed that for M-Si, the onset temperature of the oxidation reaction indicated by weight gain was advanced from 772 °C to 468 °C compared to commercially available silicon nanopowders, indicating effectively enhanced reactivity of silicon, which provides important methodological guidance for future applications of silicon-based MICs.

## 2. Results and Discussion

### 2.1. Morphology and Porosity Analysis

M-SiO_2_ was prepared by a template sol–gel process using cetyltrimethylammonium (CTA^+^) as a template surfactant, small molecule organo amines (SOA) as a mineralizing agent, and tetraethyl orthosilicate (TEOS) as an organo silica source. A high concentration of precursors with a molar composition of 1.0 SiO_2_: 0.06 CTATos: 0.026 SOA: 80.0 H_2_O was used, and the tosylate (Tos^-^) counterions favor the production of stellate porous morphology at an ultra-low SOA concentration [35]. In this process, the synergistic nucleation of surfactants and precursors in the solvent is followed by aggregation and phase separation, liquid crystal formation of inorganic molecules, and further aggregation and inorganic condensation of precursors. Finally, the desired mesoporous framework is obtained after the elimination of the template [36].

**Figure 1:**
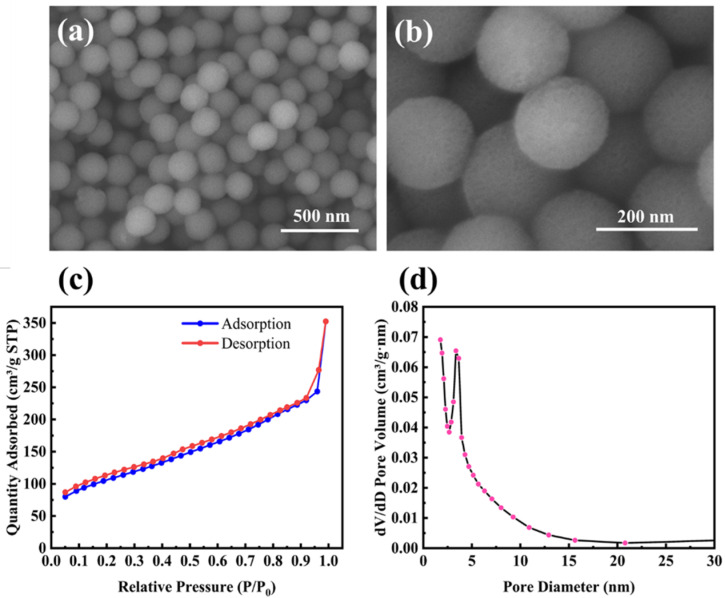
(**a**,**b**) SEM images of mesoporous silica nanoparticles (M-SiO_2_); (**c**) N_2_ adsorption/desorption isotherm; and (**d**) BJH pore size distribution curve of M-SiO_2_.

The morphology of M-SiO_2_ is shown in Figure 1a,b. It is clear that M-SiO_2_ exhibits a porous spherical structure with an average diameter of about 160 nm and a uniform size distribution. The porous properties of M-SiO_2_ were further investigated by the N_2_ adsorption/desorption isotherm, as shown in Figure 1c. The presence of an isothermal hysteresis loop was observed, which, in conjunction with the pore size distribution from 2 to 20 nm in Figure 1d, provides evidence for the presence of mesopores. The measured BET surface area was 371.7 m^2^ g^−1^, and the average pore size calculated by the BJH method was 7 nm. It should be noted that the pore size distribution shows only a narrow peak, indicating that the products are well-dispersed and do not show significant particle gaps resulting from the stacking of nanoparticles.

A magnesiothermic reduction process was used to reduce M-SiO_2_ to M-Si. In this process, the reduction reaction between magnesium and silica is highly exothermic, and the temperature generated is higher than the melting point of silicon, resulting in a large amount of fusion of the reduction products in the absence of a heat absorber, as shown in Figures S1a,b (Supplementary Materials). The melting point and the heat of fusion of sodium chloride (NaCl) are 801 °C and 28.8 kJ/mol, respectively. The melting of NaCl can bring the temperature down below the melting point of Si [37], so we introduced NaCl as a heat absorber. It is worth noting that we did not mix the solid NaCl with the other components directly; instead, we used its aqueous solution to fill the pores of M-SiO_2_ firstly and then evaporated it to crystallize, which had the advantage of allowing the NaCl to enter the pores more completely to protect the pore structure.

**Figure 2:**
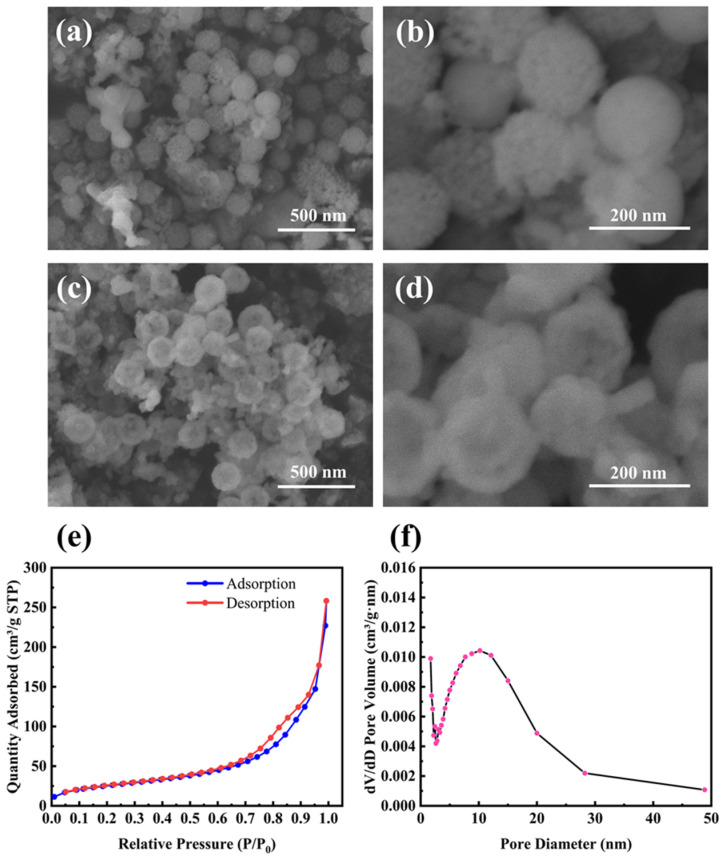
(**a**,**b**) SEM images of mesoporous silicon nanoparticles (M-Si) etched by 1 M HCl; (**c**,**d**) SEM images of M-Si etched by 1 M HCl and 5 wt% HF sequentially; (**e**) the N_2_ adsorption/desorption isotherm and (**f**) the BJH pore size distribution curve of M-Si etched by HCl and HF sequentially.

Figure 2a,b shows SEM images of M-Si etched by 1 M HCl, which was used to remove residual products such as MgO. It is clear that M-Si retains the porous structure well even after the magnesiothermic reduction treatment at 700 °C. Furthermore, the average size of the nanoparticles remains below 200 nm. Figure 2c,d shows SEM images of M-Si etched sequentially with 1 M HCl and 5 wt% HF, with the removal of unreacted SiO_2_ ensuring pure M-Si. Figure 2e,f shows the N_2_ adsorption/desorption isotherm and the BJH pore size distribution curve of the product etched by HCl and HF sequentially. The measured BET surface area was 93.0 m^2^ g^−1^, and the average pore size calculated by the BJH method was 16 nm, which was located within the mesopore range.

### 2.2. Discussion of the Magnesiothermic Reduction Process

**Figure 3:**
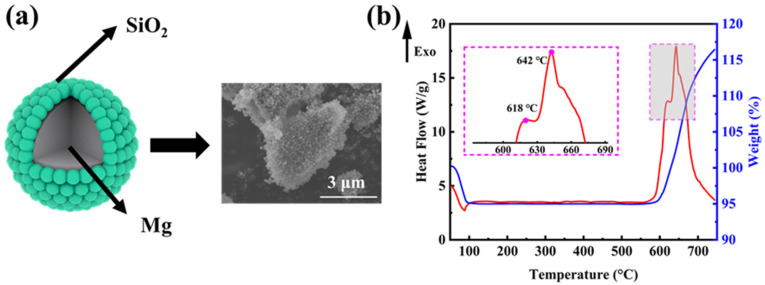
(**a**) The ideal micro-encapsulation structure between Mg powders and M-SiO_2_ and the corresponding SEM image; (**b**) the TG–DSC curve of the magnesiothermic reduction reaction in an argon atmosphere at a ramp-up rate of 10 °C min^−1^.

In order to explore the mechanism of the magnesiothermic reduction process more clearly, we investigated the weight change and exothermic properties of the ball-milled mixture (mass ratio M-SiO_2_:Mg = 1:0.8) without the addition of NaCl. Figure 3a shows the ideal micro-encapsulation structure and the corresponding SEM image, where the surface of micron-sized magnesium powder is tightly wrapped by porous silica microspheres of 160 nm in diameter. The encapsulation was realized by a ball-mill mixing method, which allows for optimized contact between the reactants and further facilitates the reaction to proceed completely. SiO_2_ undergoes the magnesiothermic reduction process as the reaction (1) [34]. The reaction (2) takes place when the amount of Mg in the reactants is excessive. By using processes (3) and (4), MgO and Mg_2_Si can be removed, respectively.

(1) $${2\mathrm{Mg}}_{\left(g\right)}+\mathrm{Si}O_{2(s)}\to {2\mathrm{MgO}}_{\left(s\right)}+{\mathrm{Si}}_{\left(s\right)}$$

(2) $${2\mathrm{Mg}}_{\left(g\right)}+{\;\mathrm{Si}}_{\left(s\right)}\to {{\mathrm{Mg}}_{2}\mathrm{Si}}_{\left(s\right)}$$

(3) $${\mathrm{MgO}}_{\left(s\right)}+{\;2\mathrm{HCl}}_{\left(l\right)}\to {\mathrm{MgCl}}_{2(s)}{+H_{2}O}_{\left(l\right)}$$

(4) $${{\mathrm{Mg}}_{2}\mathrm{Si}}_{\left(s\right)}+{\;4\mathrm{HCl}}_{\left(l\right)}\to {2\mathrm{MgCl}}_{2(s)}+{{\mathrm{SiH}}_{4}}_{\left(g\right)}$$

Figure 3b shows the DSC–TG curve for the reaction of Mg with SiO_2_ in an argon atmosphere at a ramp-up rate of 10 °C min^−1^. The weight loss before 100 °C corresponds to the detachment of adsorbed water from the reactants. At around 600 °C, there is a clear tendency for the reaction to gain weight, but theoretically, the magnesiothermic reduction reaction does not show a significant weight change. As the furnace body was not hermetically sealed, it was thought that a small amount of O_2_ leaked into the furnace during the thermal analysis. Then, prior to SiO_2_, the O_2_ reacts with the solid Mg, producing MgO shells on the surface of the Mg powders and revealing the first exothermic peak at 618 °C. With the temperature rising up, the inner core Mg starts to melt, increasing in volume and leading to the rupture of the oxide shell. The molten Mg diffuses out of the MgO shell and starts to react with SiO_2_, resulting in an increased heat release, and the second exothermic peak occurs near the melting point of Mg at 642 °C. The entire reaction is primarily concentrated within ~600–700 °C.

**Figure 4:**
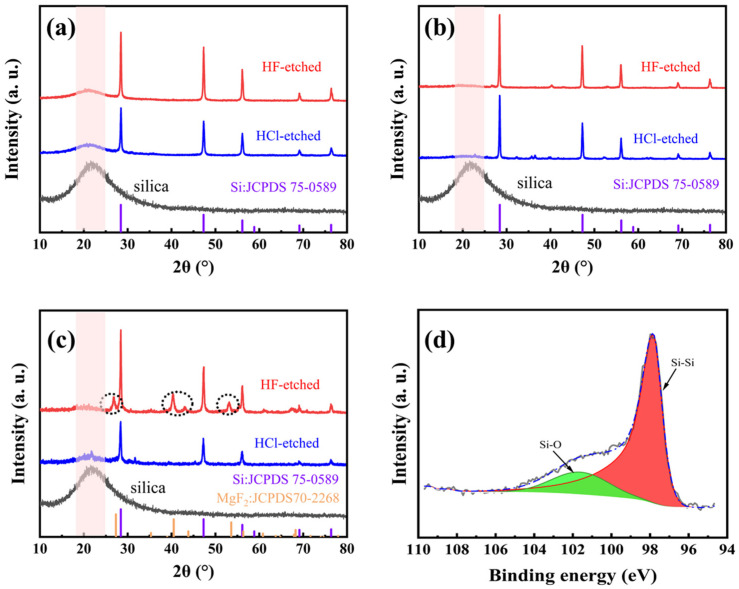
The XRD patterns of the reaction process products at different temperatures and mixing methods: (**a**) M-Si calcined at 660 °C in Ar after ball milling; (**b**) M-Si calcined at 700 °C in Ar after ball milling; (**c**) M-Si calcined at 700 °C in Ar after manual mixing. In addition, (**d**) the high-resolution XPS spectrum of Si 2p.

Besides the usage of heat absorber, both the furnace temperature and the pre-mixing methods (between M-SiO_2_ and Mg powders) have effects on the magnesiothermic reduction process. Figure 4a shows the XRD patterns of M-Si (both before and after being etched in 5 wt% HF solution) obtained at a furnace temperature of 660 °C (for 5 h), with ball milling as the pre-mixing method. The intensity of the Si diffraction peaks is enhanced after HF etching, accompanied by improved crystallinity. However, a small bulge peak near 23° attributed to amorphous SiO_2_ remains, indicating that the magnesiothermic reduction reaction does not proceed to completion. Figure 4b shows the XRD patterns of M-Si (both before and after being etched in 5 wt% HF solution) obtained at a furnace temperature of 700 °C (for 5 h), with ball milling as the pre-mixing method. It is apparent that the bulge peak due to amorphous SiO_2_ diminishes, indicating that the completeness of the reaction is improved by raising the temperature.

To simplify the process, we have also tried manual mixing besides ball milling. Figure 4c shows the XRD patterns of M-Si (both before and after being etched in 5 wt% HF solution) obtained at a furnace temperature of 700 °C (for 5 h), with manual milling as the pre-mixing method. Compared to Figure 4b, there are slight SiO_2_ amorphous peaks identified. Moreover, new diffraction peaks appear near 28°, 41°, and 53° (after being etched in 5 wt% HF solution), which are attributed to MgF_2_. The inhomogeneity of the manual mixing method results in insufficient contact of the reactants, causing a local stoichiometry unbalance and further leading to the formation of magnesium–silicon oxide, which reacts with HF to form MgF_2_. Therefore, the mixing method is also a key factor in improving the completeness of the reaction. Figure 4d shows the high-resolution XPS spectrum of Si-2p, and the peaks were corrected according to the standard peak of C 1s located at 284.8 eV. The Si-Si and Si-O signal peaks appear only at 98.1 eV and 102 eV, respectively, and the peak area ratio is 4:1, where the Si-O signal peak corresponds to the oxide layer formed on the surface of M-Si after exposure to O in air, owing to the highly reactive surface of M-Si. However, the Si-Si bond is still predominant in Si-2p, indicating that the main component is still silicon.

### 2.3. Comparison of Reactivity by Thermal Analysis

The presence of nanopores provides the particles with a huge internal specific surface area to adsorb large amounts of reactive substances, while the Si-Si bonds in these structures are more easily broken. In particular, the chemisorption that occurs on the surface of pore structures is more intense due to quantum size effects, especially for Si–H_x_ (x = 1, 2, and 3) compounds [26]. Of course, a trace amount of hydrogen molecules is also present as a result of physical adsorption. These properties allow M-Si to have a greater number of active sites and higher reactivity than currently commercially available non-porous silicon nanopowders.

**Figure 5:**
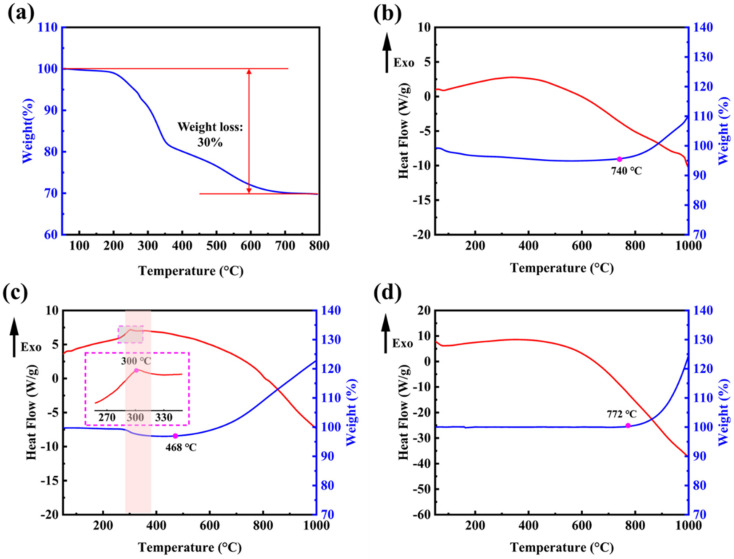
(**a**) TG curve of M-SiO_2_ before template removal; (**b**) TG–DSC curves of the newly prepared M-Si without NaCl as heat absorber; (**c**) the newly prepared M-Si with NaCl as heat absorber; (**d**) commercially available non-porous silicon nanopowders.

The remaining templates from the M-SiO_2_ preparation process can be removed by means of high-temperature calcination. In order to determine the appropriate temperature conditions, thermogravimetric tests were done on the prepared M-SiO_2_. Figure 5a shows the TG curve of the prepared M-SiO_2_ before the removal of the template; the weight loss reaction starts at 200 °C and ends at 700 °C. The whole weight loss process is mainly divided into two stages, from 200 °C to 350 °C for the fast weight-loss stage with 20% weight loss, and from 350 °C to 700 °C for the slow weight-loss stage with 10% weight loss. Figure 5b shows the TG–DSC curves of the newly prepared M-Si without NaCl as a heat absorber. Air was chosen as the furnace atmosphere to test the reactivity of both substances during heating. The oxidation process indicated by weight gain starts at 772 °C, which is only a slight advance over commercially available nonporous silica powders. Combining the fusion linkage phenomenon in Figure S1a,b, it can be seen that the disruption of the structure causes the products to lose porous properties and, thus, they have an onset temperature similar to that of nonporous silicon powders.

Figure 5c shows the TG–DSC curves of the newly prepared M-Si with NaCl as a heat absorber. In Figure 5c, M-Si shows a small exothermic peak at the low temperature section near 300 °C, which is consistent with previous reports in the literature [38]. At this temperature, it is thought that the Si–H_x_ bonds break and form Si dangling bonds and H_2_, as confirmed by the TG curve corresponding to a weight loss of 2%. The oxidation process indicated by weight gain begins almost immediately after the formation of a large number of Si dangling bonds, which are more sensitive to O_2_ molecules in air than the Si-saturated bonds of non-porous silicon nanopowders. In Figure 5d, it indicates that the commercially available silicon nanopowders started their oxidative reaction, indicated by weight gain, at 772 °C, while the temperature is 468 °C in Figure 5c. It is made possible by the large specific surface area and the rich reaction sites from the porous structure, which allows for better contact with the oxidizer.

The enhanced reactivity due to the porous structure is very attractive for the benefits of silicon-based MIC applications. The porous structure ensures that the particles are free from collapse at the nanoscale and that the oxidant can be filled into the pores in the form of a liquid phase or a solution, which results in a more uniform contact between the oxidant and the fuel and significantly reduces the mass transfer distance. In a previous literature approach [26,39], a solution of NaClO_4_ in methanol (MeOH) was dropped on the material to fill the pores in the porous silicon and act as an oxidizer. The extent of the reaction was observed by bomb calorimetry in a nitrogen (N_2_) or oxygen (O_2_) environment. In the environment without supplemental O_2_, the measured heat of the reaction was 9.9 ± 1.8 kJ g^−1^, but with supplemental O_2_, the reaction yielded 27.3 ± 3.2 kJ g^−1^, which is close to the theoretical value of 33.0 kJ g^−1^ for complete oxidation of Si by NaClO_4_. Parimi et al. prepared the porous silicon wafers using an electrochemical dissolution process and analyzed the reaction wave velocity and structure of energetic porous silicon composites formed by depositing sodium, magnesium, or calcium perchlorates within nanoscale pores [29]. Composites in the fuel-rich state, with an equivalence ratio greater than 1.60, exhibit higher temperatures and propagation velocities compared to composites with an equivalence ratio close to unity. This unusual behavior of the composites is attributed to the inhomogeneity of the system, even when the reactants are mixed on the nanoscale. The creation of porous silicon powders in powder form holds promise for further improvements in mixing homogeneity at the nanoscale.

## 3. Materials and Methods

### 3.1. Materials

Cetyltrimethylammonium tosylate (CTATos), Tris(hydroxymethyl)aminoethane (Tris) and Si nanopowders (metal basis, with the stated average diameter of 30 nm) were purchased from Marel Chemical Technology Co., Ltd., Shanghai, China. The micrographs and the N_2_ adsorption/desorption isotherm of Si nanopowders were shown in Figures S2 and S3, respectively, and a serious agglomeration phenomenon was observed, with the measured BET surface area of 19.6 m^2^ g^−1^. NaCl was purchased from Kolon Chemical Co., Ltd., Chengdu, China. Tetraethyl orthosilicate (TEOS, 98%) was purchased from Aladdin Reagents Co., Ltd., Shanghai, China. Mg powders (~100–200 mesh), HCl (~36–38%), and HF (~40%) were purchased from Sinopharm Chemical Reagents Co., Ltd., Shanghai, China. All reagents were used as received without further purification.

### 3.2. Synthesis of M-SiO_2_

M-SiO_2_ was prepared with some adaptations to the method reported previously in the literature [35]. A typical synthetic procedure is as follows. A mixture of 963 mg CTATos, 141 mg of tris base, and 50 mL of deionized water was stirred at 80 °C for 1 h. Then, 7.8 mL of TEOS was added rapidly to the surfactant solution. Note that TEOS should be poured after the surfactant has been completely dissolved in the water containing tris base. The mixture was stirred at 80 °C for a further 2 h at 1200 rpm. The product was then collected by centrifugation and washed with deionized water and ethanol. The powdered product was calcined at 700 °C for 5 h in air to remove the remaining organic template.

### 3.3. Magnesiothermic Reduction for Preparation of M-Si

**Figure 6:**
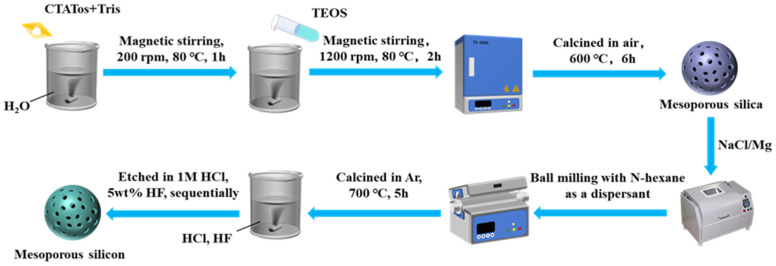
A schematic diagram of the preparation process for M-Si.

Firstly, M-SiO_2_ was added to an aqueous NaCl solution (13 wt%), where the mass of NaCl was six times that of M-SiO_2_, and the solution was sonicated for 30 min. Then, the mixture was stirred for 6 h and dried directly at 100 °C for 12 h. The products were mixed with Mg powders using a ball mill method at a mass ratio of M-SiO_2_:Mg = 1:0.8, and the mass ratio of balls to powders was set as 10:1. N-hexane was used as a dispersant in this process. For comparison, we also produced the mixtures by manual mixing. The resulting mixtures were heated in a furnace under an Ar atmosphere for reduction at controlled temperatures of 660 or 700 °C, and the reaction time was set at 5 h. After a reduction reaction, the by-products and residual SiO_2_ were removed by sequential etching with 1M HCl and 5 wt% HF. Finally, M-Si were obtained. Figure 6 shows a schematic diagram of the whole preparation process of M-Si.

### 3.4. Characterization Methods

The morphology of all samples was characterized using a scanning electron microscope (SEM, Hitachi Regulus 8100). To characterize the structural and compositional information of the samples, X-ray diffraction (XRD, Rigaku D/max-2200PC) was used, which was equipped with a Cu target (λ = 0.15406 nm, 40 kV, 30 mA, 10° min^−1^, ~10–80°, respectively). The BET-specific surface area and BJH pore size distribution of the samples were measured using an automatic surface area and porosity analyzer (Micromeritics ASAP 2460). X-ray photoelectron spectroscopy (XPS, Thermo Scientific K-Alpha, Waltham, MA, USA) was used to analyze the surface elemental composition information of the samples. The weight change and reaction onset temperature of M-Si and commercially available non-porous samples were investigated by simultaneous thermogravimetry and differential scanning calorimetry (TG-DSC, SDT-650) under programmed temperature control. During each test, the sample, with a mass of roughly 2 mg, was placed into an alumina crucible and heated in flowing air (50 mL min^−1^) from 50 to 1000 °C at a ramp-up rate of 10 °C min^−1^.

## 4. Conclusions

We have demonstrated that M-Si could be obtained using a molten salt-assisted magnesiothermic reduction process. For M-Si made by this method, the onset temperature of the oxidation reaction indicated by the weight gain is ~468 °C, which is ~304 °C lower than that of commercially available non-porous nanopowders. The lower reaction onset temperature is due to the fact that the porous structure provides the particles with a large internal specific surface area, resulting in unique interfacial properties and reduced mass diffusion limitations. Together with the abundant reactive sites that make M-Si sensitive to O_2_ molecules in air, they enhance the reactivity of silicon as a fuel candidate.

## Data Availability

Not applicable.

## Supplementary Materials

The following supporting information can be downloaded at: https://www.mdpi.com/article/10.3390/molecules28073274/s1, Figure S1: SEM images of M-Si by a magnesiothermic reduction process without a heat absorber; Figure S2: SEM images of commercially available non-porous silicon nanopowders; Figure S3: The N_2_ adsorption/desorption isotherm of commercially available non-porous silicon nanopowders.

## Sample Availability

Samples of the compounds are available from the authors.
