# Supplementary material for: Preparation of Mesoporous Si Nanoparticles by Magnesiothermic Reduction for the Enhanced Reactivity
Source: Molecules. 2023 Apr 6;28(7):3274. doi: 10.3390/molecules28073274 (PMC10096974; doi:10.3390/molecules28073274)
Supplement: Supplementary file 1 [file molecules-28-03274-s001.zip › molecules-2309357-supplementary.pdf]

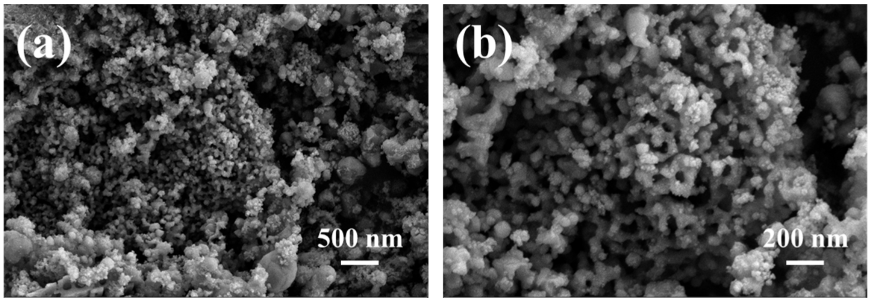

Figure S1. SEM images of M-Si obtained through a magnesiothermic reduction process without the use of a heat absorber.

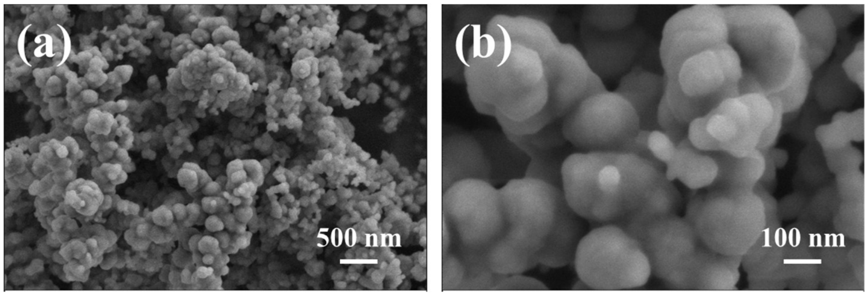

Figure S2. SEM images of commercially available non-porous silicon nanopowders.

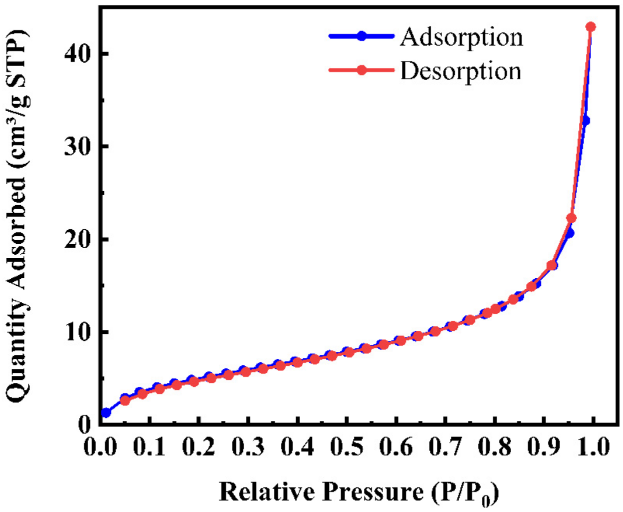

Figure S3. The N_2 adsorption/desorption isotherm of commercially available non-porous silicon nanopowders.
